# Acinic Cell Carcinoma of Minor Salivary Gland of the Base of Tongue That Required Reconstructive Surgery

**DOI:** 10.1155/2012/421065

**Published:** 2012-12-12

**Authors:** Kota Wada, Subaru Watanabe, Yuji Ando, Yoichi Seino, Hiroshi Moriyama

**Affiliations:** ^1^Department of Otolaryngology, Asahi General Hospital, Chiba 289-2511, Japan; ^2^Department of Otorhinolaryngology—Head and Neck Surgery, Jikei University School of Medicine, Tokyo 105-8461, Japan

## Abstract

Acinic cell carcinoma of minor salivary gland of the base of tongue is very rare. Squamous cell carcinoma is the most common tumor in the base of tongue. We present a patient with gigantic acinic cell carcinoma of the base of tongue. This patient required emergency tracheotomy before surgery, because he had dyspnea when he came to our hospital. We removed this tumor by pull-through method and performed reconstructive surgery using a rectus abdominis myocutaneous flap. It was a case that to preserved movement of the tongue and swallowing function by keeping lingual arteries and hypoglossal nerves. This case was an extremely rare case of ACC of the base of tongue that required reconstructive surgery.

## 1. Introduction

Primary neoplasms of minor salivary gland tissue are not common in addition neoplasm arising in minor salivary gland of the base of tongue is very rare. Squamous cell carcinoma is the most common tumor in the base of tongue [1]. Acinic cell carcinoma (ACC) of minor salivary gland of the base of an tongue is extremely rare tumor. ACCs around head and neck area are normally found in parotid gland [2, 3]. ACC is a malignant epithelial neoplasm in which the neoplastic cells demonstrate acinar differentiation [4]. 

We present the case of gigantic ACC of minor salivary gland of the base of tongue. This case required reconstructive surgery, because the resection resulted in a large deficit. We removed this tumor by pull-through method and performed reconstructive surgery using a rectus abdominis myocutaneous flap. Because we kept lingual arteries, hypoglossal nerves, it was a case that preserved movement of the tongue and swallowing function. This case was an extremely rare case of ACC of the base of tongue that required reconstructive surgery.

## 2. Case Presentation

**Figure 1:**
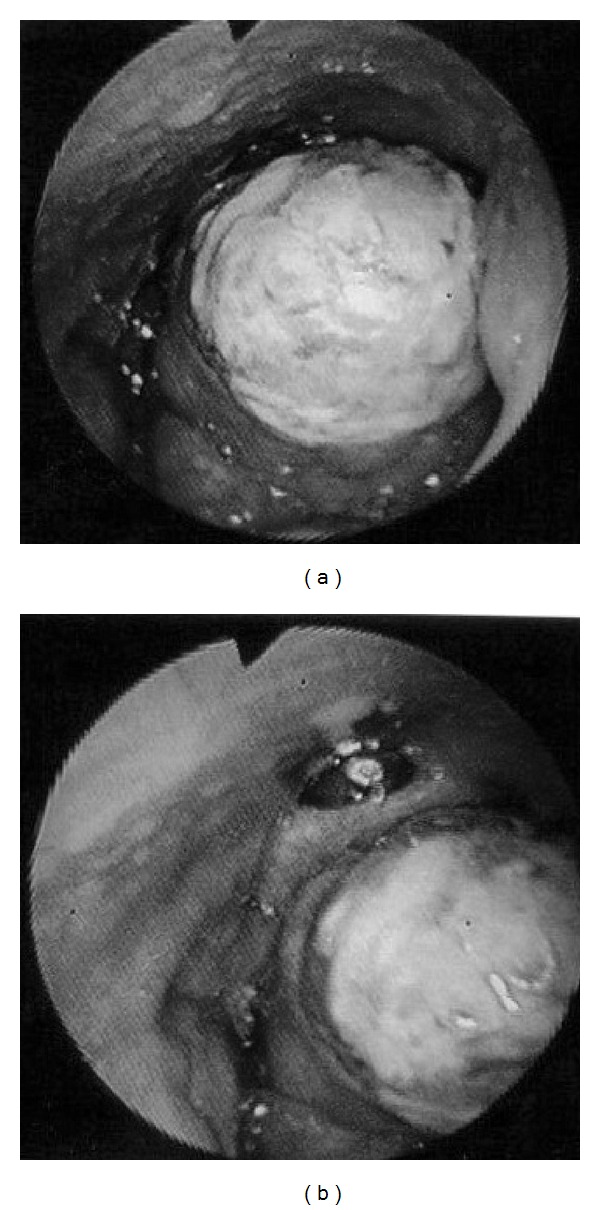
(a) A gigantic tumor, with necrosis on its surface, is seen near the center of the base of the tongue. (b) The airway is almost blockaded and epiglottis is oppressed backward by the tumor.

**Figure 2:**
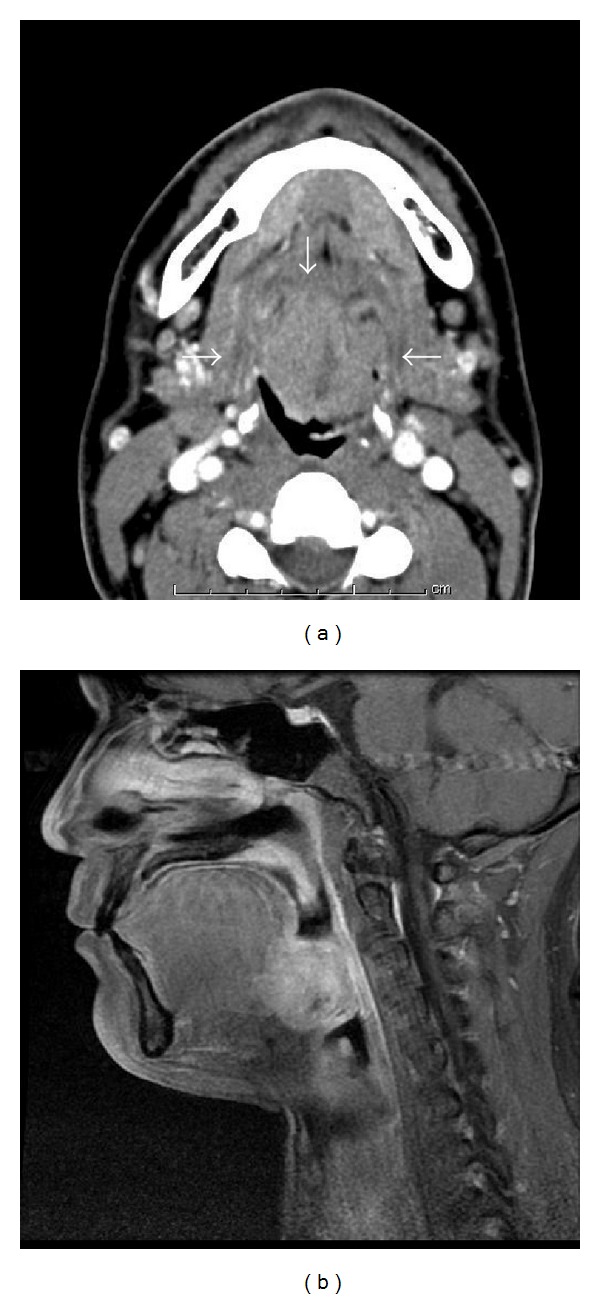
(a) An invasive tumor with a flat and smooth surface is seen at the midline of the base of the tongue (axial view of CT). (b) Enhanced MRI reveals a well-contrasted tumor, while the lower portion of the oropharynx is nearly closed (sagittal view of MRI).

**Figure 3:**
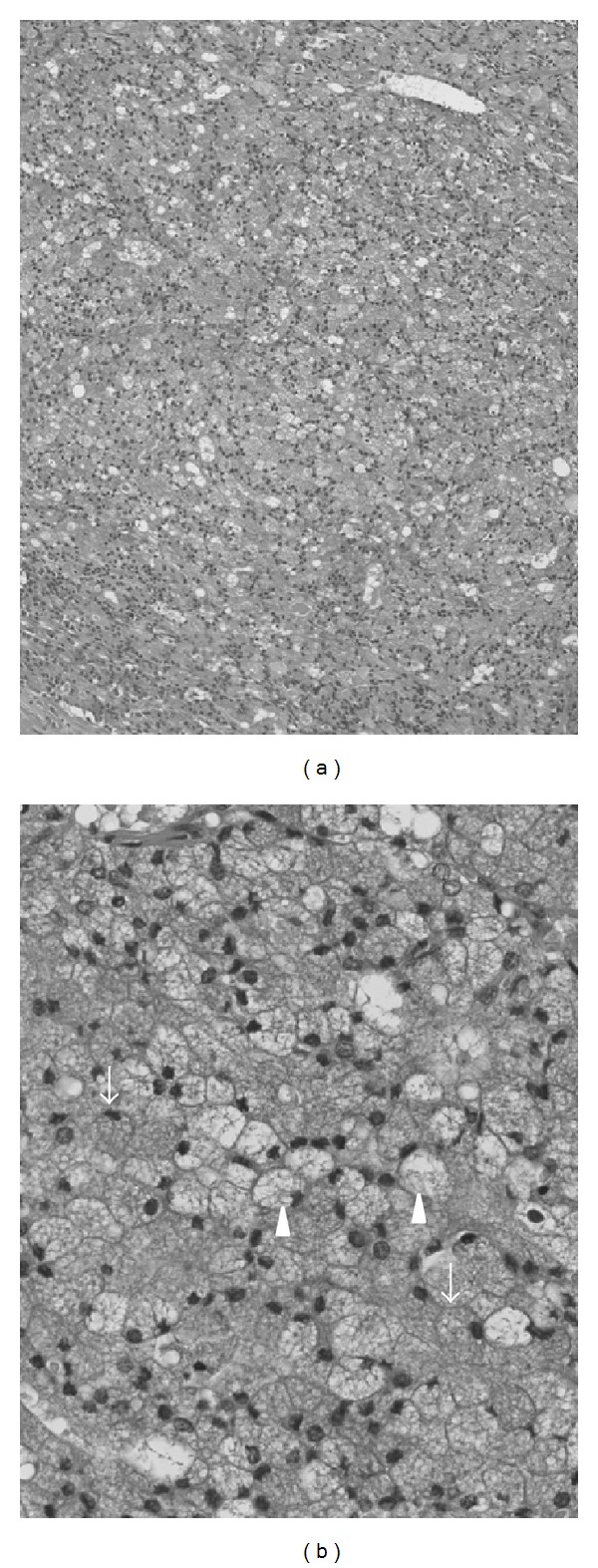
(a) The acinar cells have proliferated in sheet-form, and the tumor is classified as the solid-growth pattern (H&E stain, original magnification 100X). (b) An acinar cell (arrow) shows violet staining of the cytoplasm and an eccentrically positioned nucleus, while the cytoplasm of a vacuolated cell (arrowhead) is seen to be filled with vacuoles (H&E stain, original magnification 400X).

**Figure 4:**
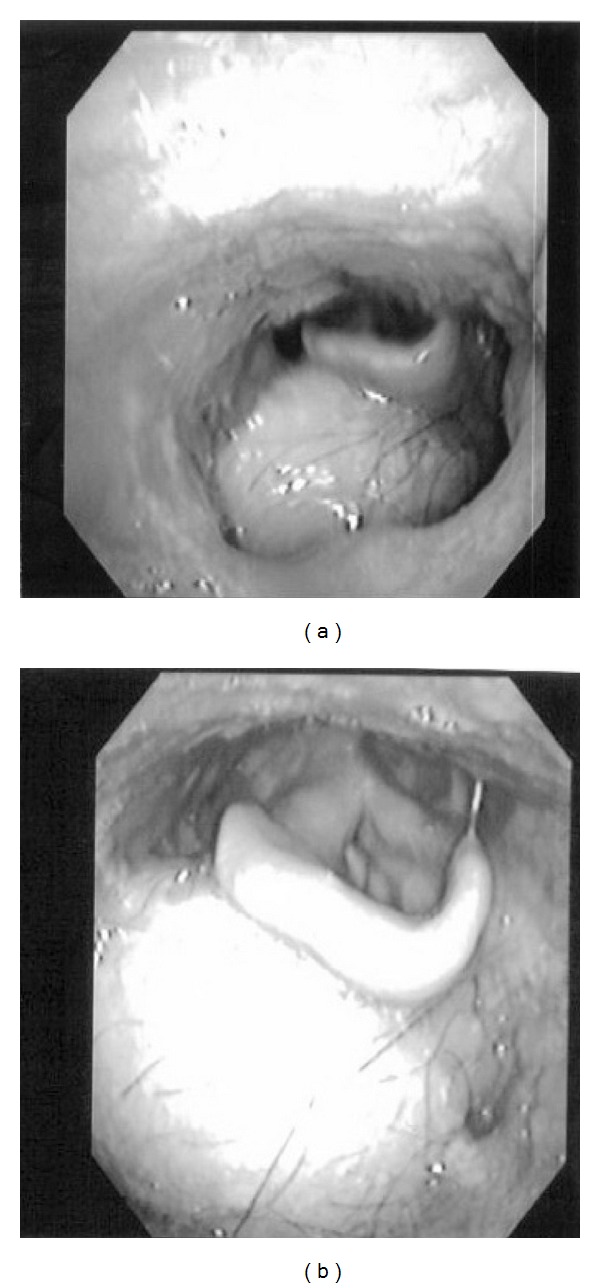
(a) The base of tongue was reconstructed by using a rectus abdominis myocutaneous flap. In this case of reconstructive surgery, we formed the base of tongue by heaping up the base of tongue and raised larynx also for function of swallowing. (b) The airway is well-maintained after resection and reconstructive surgery.

A 32-year-old man presented to our hospital with a sore throat, obstruction around the larynx and dyspnea. He had no specific past medical history or history of smoking. Laryngeal fiberscopic examination showed a large mass with necrosis at the middle of the base of tongue (Figure 1(a)). Movement of the tongue was normal, but the airway was almost completely blocked (Figure 1(b)). Palpation of the cervical region did not detect lymphadenopathy. Contrast-enhanced computed tomography (CT) demonstrated a 30-mm solid and well-defined mass involving the whole base of tongue (Figure 2(a)). The sagittal view of magnetic resonance imaging (MRI) identified a 34-mm mass occupying the pharyngeal space with a high intensity signal on T2 sequence and low intensity on T1 (Figure 2(b)). Emergency tracheotomy was performed under local anesthesia, and biopsy was also performed after management of the airway. Histopathological studies showed differentiation to serous cells, while there was no existing glottal structure or fibrous stroma, and proliferative lesions having an acinous structure were seen. No cellular atypia or nuclear division was observed, but partial surface necrosis and vascular infiltration were seen. PAS staining and PAS digestive staining (i.e., diastase treatment followed by PAS) showed positive staining of the cytoplasm of the tumor cells. Mucicarmine staining was negative, showing zymogen-like granules, not mucinous granules. The findings indicated an epithelial malignant tumor showing an acinar or microcystic pattern of invasive growth, and a diagnosis of an oropharyngeal acinar cell adenocarcinoma was made. Resection of the tumor and conservative neck dissection were performed in Jikei University Hospital. This tumor was resected by pull-through method. The tumor resection resulted in a large deficit, and reconstructive surgery was performed using a rectus abdominis myocutaneous flap. For function of swallowing and movement of tongue, we preserved tongue and larynx by keeping lingual artery and hypoglossal nerve. The resected tumor was gray-white, 40 × 33 × 25 mm in size and elastic-hard. Histopathological specimens of the resected tumor showed no contradictions of the preoperative findings and no necrosis inside the tumor (Figure 3). Special staining was performed by PAS and digestive PAS, and the results indicated the tumor to be positive for each of CA125, CK7, CD15 (Leu-M1 antigen), alpha-1-antichimotrypsin, Mib1 (Ki-56: 5%), and p53 (2%), and negative for AluBlue, CA19-9, CK20, AT, and GFAP. There was no lymph node metastasis. Postoperatively, the functions of swallowing and speech were very good and there has been no sign of recurrence or metastasis as of two years of followup (Figure 4).

## 3. Discussion

Acinar cell carcinoma (ACC) is a salivary gland tumor that is characterized by proliferation of cells resembling serous acinar cells [4], and ACC is said to constitute 6~10% of all salivary gland tumors [5, 6]. They most commonly occur in patients 30~40 years of age and predominantly in women [7]. These carcinomas originate in a parotid gland in 86.3% of patients, a submaxillary gland in 2.7%, and a minor salivary gland in 9%. When the origin is a minor salivary gland, the buccal mucosa and labial and palatal glands are most frequently involved, whereas origin in the glossal glands is rare [3, 7]. To date, ACC originating in the tongue have involved the dorsal surface of the tongue or the lateral portion of the base of tongue [8, 9]. The patient reported here had presented with formation of a large tumor at the center of the base of tongue and dyspnea, and it was a rare case that required tracheotomy. In addition, the surgical resection of this patient's tumor made it necessary to perform reconstructive surgery. There is a lot of argument regarding pull-through method [10–12], we chose pull-through method, because the tumor was present in the base of tongue and size was gigantic. We preserved larynx and most of tongue for function of swallowing by keeping lingual arteries and hypoglossal nerves. In this case of reconstructive surgery, we formed the base of tongue by heaping up the base of tongue and raised larynx also for function of swallowing. But, surgery for neoplasm of the base of tongue is very difficult. In this case, the result of pathology was ACC, not invasive type of carcinoma and surgical resection was only therapy for this patient, then we selected this method. We need to increase cases in future and have to establish the therapy for neoplasm of the base of tongue. 

The neoplasm of salivary glands can be distinguished from normal salivary glands because it has an unclear acinous structure and lack intercalating ducts. ACC is defined as a tumor that has differentiated from cells resembling serous acinar cells in a normal salivary gland, and the cells contain zymogen granules as secretory granules. ACC is further characterized by containing cytoplasmic granules (zymogen granules) that are positive in PAS staining, positive diastase-treatment PAS staining and negative in mucicarmine staining [3, 4]. The findings for the present patient conformed with those characteristics. The histological architecture of ACC is diverse, and Abrams et al. have proposed a classification consisting of five histological and five cell subtypes [12]. The histological subtypes are (1) solid pattern, (2) microcystic pattern, (3) papillary-cystic pattern, (4) follicular pattern and (5) adenomatous pattern. Similarly, the cell subtypes are (1) acinar cells, (2) intercalated ductal cells, (3) vacuolated cells, (4) clear cells, and (5) nonspecific glandular cells. The present patient's ACC was judged to be the solid pattern because it had proliferated in a sheet and alveolar configuration (Figure 3(a)). Also, as its cell subtype, it was observed to consist partially of vacuolated cells, although it was judged to be the acinar cell subtype because the predominant component consisted of tumor cells having a slightly basophilic, violet-stained cytoplasm and a nucleus that was oval, eccentrically located and darkly stained (Figure 3(b)). Immunohistochemically, the cells stained positively for cytokeratin, transferrin, alpha-1-antitrypsin, alpha-1-antichymotrypsin, IgA, Leu-M1, lactoferrin, CEA, and S-100 protein, although none was specific [3]. The findings of positive staining for cytokeratin and CEA are suggestive of differentiation to ductal epithelium. 

As a differential diagnosis, the other possibilities include epithelial myoepithelial carcinoma, mucoepidermoid carcinoma accompanied by oncocytic change, oncocytoma, multicellular pleomorphic adenoma, Warthin's tumor, metastatic renal cell carcinoma and sialoadenosis. It is necessary to differentiate cases with numerous vacuolated cells or the cystic type from adenoid cystic carcinoma, [13–15]. Also, although the papillary-cystic pattern is rare, it is important to differentiate it from metastasis of thyroid cancer [16]. 

The 5-year survival rate of ACC is relatively good at approximately 80% [2, 5]. Factors leading to a poor prognosis for ACC are said to include (1) short duration of symptoms, (2) incomplete excision, (3) frequent mitoses, (4) focal necrosis, (5) neural invasion, (6) pleomorphism, (7) infiltration, and (8) stromal hyalinization. In particular, the prognosis is extremely poor in the case of dedifferentiated ACC, in which the ACC is accompanied by a highly malignant adenoma, poorly differentiated carcinoma, or undifferentiated carcinoma. It was also reported that the prognosis is poor in cases with an MIB-1 index (Ki-67) of 5% or more [17]. The ACC of this patient we have reported showed little cellular atypia, but the duration from onset was unclear, it was expanding with an unclear border relative to the stroma, there was vascular infiltration (although slight) and there was partial necrosis. The patient was monitored for two years after the surgical resection of the ACC, and there were no signs of recurrence of the malignancy. However, continued careful followup of this patient will be required in light of the fact that have been reports of cases of recurrence of ACC even after more than 10 years [18].

## 4. Conclusion

Acinic cell carcinoma from minor salivary gland is not common. Especially, acinic cell carcinoma of the base of tongue is extremely rare. We reported here a patient with a giant acinic cell carcinoma developed at the base of tongue. This case was a rare case that had been complicated by dyspnea. We removed this tumor by pull-through method and performed reconstructive surgery.
